# Understanding the Connection between Cognitive Impairment and Mobility: What Can Be Gained from Neuropsychological Assessment?

**DOI:** 10.1155/2017/4516219

**Published:** 2017-04-27

**Authors:** Marykay A. Pavol, Joel Stein, Foyruz M. Kabir, Jonathan Yip, Lyssa Y. Sorkin, Randolph S. Marshall, Ronald M. Lazar

**Affiliations:** ^1^Stroke Division, Department of Neurology, Columbia University College of Physicians & Surgeons, New York, NY, USA; ^2^Department of Rehabilitation and Regenerative Medicine, Columbia University College of Physicians & Surgeons, New York, NY, USA; ^3^Department of Biostatistics, Mailman School of Public Health, Columbia University, New York, NY, USA

## Abstract

The ability of neuropsychological tests to predict rehabilitation outcome is unclear, particularly when other ratings of cognition are available. Neuropsychological test scores and functional ratings of cognition (Functional Independence Measure (FIM) Cognition score) were used to predict improvement in patient mobility and self-care skill, as measured by the FIM Motor score. Regression models used both raw neuropsychology test scores and age-adjusted scores. Retrospective chart review was performed for patients on an inpatient rehabilitation unit and referred for neuropsychological assessment. The group included 126 subjects (average age 64.2 ± 17.1 years) and a variety of medical diagnoses. Neuropsychological tests included the Repeatable Battery for the Assessment of Neuropsychological Status (RBANS). After forcing the Admission FIM Cognition score into the model, RBANS scores and duration of rehabilitation predicted FIM Motor improvements (*F* = 11.42, *p* < 0.0001). Raw neuropsychological test scores performed better than the model with age-adjusted test scores. FIM Cognition alone did not predict FIM Motor improvements. Neuropsychological tests, combined with duration of rehabilitation, predicted mobility gains for patients undergoing inpatient rehabilitation beyond what was predicted by another, readily available, assessment of cognition. Neuropsychology raw scores performed better than age-adjusted scores, raising questions about the standard use of demographic adjustments for predicting real-world function.

## 1. Introduction

For patients receiving inpatient rehabilitation, cognitive impairment has been demonstrated to have a negative effect on self-care skills [1–5]. Information about a patient's cognitive skills can aid in the prediction of rehabilitation outcome [6–12] and greater knowledge of this predictive relationship would be useful in anticipating patient assistance needs at discharge. Information about cognition is routinely obtained during the rehabilitation admission through ratings by rehabilitation therapists. These ratings relate to cognition in a functional, applied setting. In addition, some patients are referred for neuropsychological exam during the rehabilitation admission but it is unclear whether this additional exam provides unique information in predicting self-care and mobility skills. We examined whether mobility gains for patients with cognitive deficit could be predicted from neuropsychological test scores* beyond* what is predicted by readily available, functional ratings of cognition.

Mobility and self-care skills were assessed with the Functional Independence Measure (FIM) [13], one of the most common measures of functional ability, and typically used with patients undergoing inpatient rehabilitation. The mobility and ADL items of the FIM are typically combined to produce the Motor subscale (e.g., walking, transferring to/from a wheelchair, dressing, and toileting) and the remaining items form the Cognitive subscale (e.g., memory, communication, and social skills) [14]. FIM ratings represent a subjective estimate of the amount of assistance the patient requires to complete a task. An association between Motor and Cognitive FIM subscales has been found in patients with traumatic brain injury [15, 16], cardiac arrest [17], and stroke [18, 19].

Neuropsychological assessment is another method of obtaining information about cognition. The neuropsychological exam involves standardized administration and interpretation of cognitive tests through objective methods. While informative, the neuropsychological assessment requires a separate evaluation and is not routinely performed for rehabilitation patients. A brief neuropsychological instrument, the Repeatable Battery for the Assessment of Neuropsychological Status (RBANS) [20], is well suited to the inpatient rehabilitation setting. The RBANS consists of subtests that evaluate memory, attention, language, and visual spatial skills. The RBANS has been found to predict functional outcome for a variety of patient diagnoses [3, 21–23].

The current study explored whether the* addition* of neuropsychological (RBANS) scores improved prediction of self-care/mobility skills (Motor FIM) beyond what was predicted by the Cognitive FIM for patients with suspected cognitive impairment [24]. Specifically, this study used a multiple regression approach to predict change in mobility during rehabilitation and forced the Cognitive FIM into the model first; RBANS variables would remain in the regression model only if they added to prediction of mobility change* after* the contribution of the Cognitive FIM variable.

Lastly, neuropsychological test scores are typically interpreted by making comparisons to normative groups that are demographically similar to the patient in question (e.g., comparing patient scores to a normative sample similar in age). These calculations provide information about a patient's abilities relative to a specific peer group. Two prior reports, however, support the use of unadjusted (raw) neuropsychological scores when addressing questions about a patient's function in the “real world” [25, 26]. The unadjusted scores provide information about absolute skill level, regardless of demographic factors. We therefore examined whether RBANS scores that were* unadjusted* for age (raw scores) better predicted change in mobility and ADL function than those that were age-adjusted (*z*-scores).

## 2. Methods

### 2.1. Subjects

The study was performed at an urban teaching hospital. The study was approved by the Institutional Review Board prior to data collection and all procedures were consistent with ethical standards. Data were collected retrospectively for patients admitted to an inpatient acute rehabilitation unit. Patients were admitted with a range of medical diagnoses (neurologic and nonneurologic). Criteria for rehabilitation admission included documented assistance needs (e.g., ambulation, self-care, and/or cognition), active insurance, and medical stability. Criteria for rehabilitation discharge included achievement of rehabilitation goals, inability to tolerate or benefit from therapy, or medical decompensation. Subjects were seen for neuropsychological assessment following clinical referral from the attending physician to characterize suspected cognitive deficit and/or for discharge planning purposes. Study inclusion criteria for the neuropsychology assessment group included the following. (1) An RBANS exam deemed valid without significant concerns about effort or hearing. Poor effort was defined as an RBANS Effort Index score greater than 3 [27] or statements from the subject that he/she did not wish to participate in the evaluation. Subjects with visual or motor deficits were included if the subject was able to perform verbal measures. (2) The subject was fluent in English and reported English as the preferred language. (3) The subject was not aphasic. (4) The subject had a rehabilitation admission of at least two days. (5) Most verbal tests were completed. If the subject underwent assessment during more than one admission, the data from the first exam were used. FIM data included Admission and Discharge FIM scores and medical diagnosis. The difference between the Admission and Discharge Motor FIM scores is the Motor FIM Gain. All medical diagnoses were included. The length-of-stay (number of rehabilitation days) and the RBANS assessment date were also considered as variables for prediction of FIM Motor Gain.

### 2.2. Procedures

Self-care, mobility, and cognition were assessed by rehabilitation therapists through use of the FIM [13]. The FIM includes 18 items, 13 for the assessment of mobility and basic activities of daily living (ADL) (e.g., eating, grooming, and ambulation) and five for the assessment of cognitive and communication skills (comprehension, expression, social interaction, problem-solving, and memory). Ratings are based on observation of the amount of assistance needed to complete an activity and higher FIM scores indicate greater functional independence. The Motor FIM score was comprised of the 13 mobility and ADL items whereas the Cognitive FIM score was comprised of the 5 items that rated communication, memory, and social interaction. Neuropsychological testing was performed by a board-certified neuropsychologist. RBANS raw scores and age-adjusted *z*-scores were calculated for each subtest according to normative data from the test publisher [20]. Higher RBANS scores indicate better ability. RBANS subtest scores were used instead of Index scores in order to minimize missing data because visual/motor deficits prevented some patients from completing all subtests, thus prohibiting calculation of Index scores for some patients. Nine of the 12 RBANS subtests were included: Figure Copy, Line Orientation, Semantic Fluency, Digit Span, Coding, List Recall, List Recognition, Story Recall, and Figure Recall. Other subtests were omitted to reduce the number of variables and because of collinearity (Immediate Memory subtests) and skewing (Naming subtest).

### 2.3. Statistics

Statistical analyses were performed with SAS software [28]. A stepwise approach was used for model-building purposes, due to the minimal prior research available to guide selection of RBANS subtests. First, univariate analyses were used to identify predictors to incorporate into a multivariate model. Plots of significant univariate predictors were created to determine linearity with the FIM Motor score variable. Correlations among significant univariate predictors were also examined to assess for multicollinearity and to aid in model building. Variables that were statistically significant in the univariate tests were included in multiple regression models to predict FIM Motor Gain. *t*-tests were performed to compare the Admission Motor FIM and Admission Cognitive FIM scores between the subjects in the neuropsychology study group and the subjects who underwent rehabilitation during the same time period but were not referred for neuropsychological assessment.

The RBANS scores found to be significant in the univariate tests were used to predict FIM Motor Gain scores after forcing the Admission Cognition FIM score into the model. Regression analyses were performed for both RBANS raw score and age-adjusted* z*-score models. Patient age was intentionally not used as a covariate in order to compare models that used age-adjusted* z*-scores against models that did not adjust for age. Manual backward selection was utilized to create the best final model. After the best model was identified, residual diagnostics were completed to assess for violations of the assumptions for linear regression. The Akaike Information Criterion (AIC) test was done to compare the raw score and* z*-score regression models [29].

To examine the influence of medical diagnosis on FIM Motor Gain, subjects were divided into two groups according to whether or not the primary admission diagnosis was associated with brain dysfunction. All diagnoses with CNS involvement were in the brain injury group, except those with only spinal cord involvement. An ANCOVA was performed to compare FIM Motor Gain scores between the two diagnosis groups, with age as a covariate.

## 3. Results

Data were collected from January 2008 through August 2011, during which time there were 1543 rehabilitation admissions; 134 patients underwent neuropsychological exams in English; 4 patients were excluded due to aphasia, 2 patients were excluded due to rehabilitation admission duration under 2 days, and 2 patients were excluded due to substantial missing data. The final study sample included 126 subjects with an average age of 64.2 (17.1) years, average education of 13.8 (3.7) years, and 55 women (44%). The average duration of rehabilitation admission was 12.7 (6.8) days. The sample included a range of medical diagnoses (see Table 1). Impairments were found in all cognitive domains except for auditory attention, as demonstrated by mean *z*-scores for the RBANS subtests (see Table 2).

The *t*-tests comparing groups that did (*n* = 126) and did not (*n* = 1409) undergo neuropsychological exam found no differences in the Admission Motor FIM scores (*p* = 0.44). The group that was referred for neuropsychological exam had, however, significantly lower Admission Cognitive FIM scores (*p* = 0.0002).

The length-of-stay (number of rehabilitation days) variable was found to correlate with FIM Motor Gain and was therefore included as a predictor in the regression models (*r* = 0.23, *p* = 0.0102). The RBANS assessment date variable did not significantly correlate with FIM Motor Gain and thus was not included in the regression models (*r* = 0.15, *p* = 0.1016).

After forcing the Admission Cognition FIM score into the regression equation, some RBANS scores were significant predictors of Motor FIM Gain. The best raw score model for predicting Motor FIM Gain included the Admission Cognition FIM, RBANS List Recognition, RBANS Figure Recall, and length-of-stay variables (see Table 3). The best *z*-score model included Admission Cognition FIM, RBANS List Recognition, and length-of-stay scores. The AIC test showed that the raw score model performed better than the *z*-score model and accounted for nearly 30% of the variance. Higher RBANS scores and length-of-stay durations were associated with greater Motor FIM Gain. Without the addition of the RBANS scores, the Admission Cognitive FIM score did not predict Motor FIM Gain (*p* = 0.07).

Subjects were divided into groups of patients with and without brain dysfunction as the primary diagnosis. An ANCOVA (controlling for age) was performed to compare Motor FIM Gain for the two groups. There were no significant group differences (With Brain Dysfunction group *n* = 91, Mean Motor FIM Gain = 25.95; Without Brain Dysfunction group *n* = 35, Mean Motor FIM Gain = 25.06, Pr > *F* = 0.3896).

## 4. Discussion

In this selected group of patients with suspected cognitive deficit and referred for neuropsychological assessment, RBANS scores were found to add to the prediction of FIM Motor improvements* after* inclusion of the FIM Cognitive score in the regression model. The Cognitive FIM score alone did not predict gains in mobility and ADLs. The duration of rehabilitation (“length-of-stay”) was also, not surprisingly, a significant predictor of FIM Motor Gain. Nevertheless, even when including the length-of-stay variable in the regression model, RBANS scores significantly added to prediction of FIM Motor Gain. These results support the use of standardized neuropsychological assessment in predicting outcome for patients undergoing inpatient rehabilitation. The study group referred for neuropsychological exam did not differ in Admission Motor FIM scores compared to the group that was not referred, demonstrating that functional motor skills did not differ between the groups at the start of rehabilitation therapies. The finding that these groups did differ in Admission Cognitive FIM scores, with lower Cognitive FIM scores for the group referred for neuropsychological exam, supports the rationale for referrals for neuropsychological exam but also indicates that our findings cannot be generalized to all rehabilitation patients.

An association between cognition and FIM scores (Motor and Cognitive subscales) has previously been reported for patients with traumatic brain injury [15, 16], cardiac arrest [17], and stroke [18, 19]. In studies of stroke patients, improvement in FIM mobility scores (FIM Motor Gain) has been associated with higher cognitive function [8, 10–12, 30]. Hip fracture patients undergoing inpatient rehabilitation showed a correlation between FIM Motor Gain and a baseline mental status exam score [9]. In a study with mixed patient diagnoses, Barnes and colleagues found that patients with more cognitive impairment showed less FIM Motor Gain [6]. Cullen and Weisz reported that patients with anoxic brain injury achieved lower FIM Motor Gains than patients with traumatic brain injury, and several neuropsychological tests correlated with the functional outcome [7]. Our findings are consistent with this body of work.

Our findings suggest that the Cognitive FIM score cannot substitute for a neuropsychology exam in predicting rehabilitation mobility outcome. The reason for the failure of the Cognitive FIM score, used in isolation, to predict the FIM mobility gains in this sample is unclear. The FIM Cognitive score has been studied as a correlate of the FIM Motor score and as compared to other cognitive tests, and the findings have been mixed. The Admission Cognitive FIM score has shown some ability to predict improvements in the Motor FIM score [6, 18, 30]. Some studies have reported a significant association between the Admission Cognitive FIM score and other measures of cognition [8, 15]. Conversely, other studies found little to no relationship between Cognitive FIM scores and other cognitive test scores [31, 32]. The current findings may be the result of the differences in the nature of the Cognitive FIM and the neuropsychological assessment, because the Cognitive FIM relies on subjective ratings by therapy staff, as opposed to the objective, standardized data collected from the neuropsychology exam. Additionally, the Cognitive FIM score comprises ordinal rankings of cognitive ability, with potential problems with “floor” and “ceiling” in ratings, whereas neuropsychological scores (raw and *z*-scores) are continuous measures, allowing for greater sensitivity to differences in performance.

The RBANS has a history of use in inpatient rehabilitation units. Gordon et al. reported that RBANS scores (total score and subtest scores) predicted Motor and Cognitive FIM scores in patients undergoing inpatient rehabilitation for a variety of diagnoses [21]. Larson and colleagues used the RBANS in the assessment of stroke patients undergoing inpatient rehabilitation [22, 23]. They found that the RBANS total and Index scores predicted the Cognitive FIM score at 6 months and 12 months but did not predict the Motor FIM score at either follow-up exam. A study of patients in a memory disorders clinic found that performance on the RBANS (subtest scores) was significantly associated with informant report of functional ability [3]. Ours is the first study to show that neuropsychological scores can predict mobility gains,* after* accounting for the influence of the Cognitive FIM rating. The reasons for the strength of specific RBANS subtests in the regression models (e.g., List Recognition and Figure Recall) are unclear and since an exploratory stepwise approach was used, the results must be viewed with caution. Nonetheless, there is evidence that tests of delayed memory, such as RBANS List Recognition and Figure Recall, may be among the most sensitive measures of brain dysfunction [33, 34].

Our results also showed that the model with RBANS* raw* scores predicted FIM Motor Gain better than the model which used* age-adjusted z*-scores. Patient age was intentionally not used as a covariate in order to compare models that used age-adjusted *z*-scores against raw score models that did not adjust for age. This finding raises questions about whether the practice of “correcting” neuropsychology scores according to demographic characteristics (e.g., age, education, and race) is the best method of predicting self-care/mobility. The issue of demographic adjustment for neuropsychological scores has been discussed in detail by Silverberg and Millis in 2009 [25] and Barrash et al. in 2010 [26]. Silverberg and Millis made a distinction between “impairment” (whether a patient has cognitive decline compared to a reference group) and “deficiency” (whether the patient has cognitive difficulties sufficient enough to interfere with functional task performance in the real world). Assessment of “impairment” requires the comparison of a patient's raw score to expected scores, based on age and other relevant demographic factors. In contrast, assessment of “deficiency” requires comparison of the patient's raw score to that of a healthy adult sample, regardless of demographic factors. In other words, to examine “deficiency” there is no “correction” for older age, lower education, or other factors that may influence performance. Both the 2009 and 2010 papers contend that, for questions regarding ability to perform a functional task, it is more appropriate* not *to use demographically corrected scores, as “it is the examinee's absolute levels of relevant ability that are pertinent.” [26] The implication is that, for questions about real-world function, there is evidence that neuropsychological raw scores are the most appropriate measure, a position that is supported by our findings, because our model with raw scores showed better predictive ability than the model with scores adjusted for age.

The current study found that subjects with and without diagnoses that involve brain dysfunction achieved the same degree of improvement over the course of their rehabilitation. This suggests that projection about a patient's ability to benefit from rehabilitation therapies should not be based on diagnosis alone. Factors other than diagnosis, such as presence of medical comorbidities, severity of physical deficits, and patient demographics, may have greater influence rehabilitation outcome [35–37].

### 4.1. Study Limitations

The stepwise statistical methods used in this study are most appropriate for model building; thus the predictive accuracy of these models must be verified before firm conclusions can be drawn. Other limitations include the small sample size and the retrospective nature of the analyses. The subjects in our sample had significantly lower Admission Cognitive FIM scores than those who were not referred for neuropsychological assessment. These limitations create selection bias and reduce the generalizability of the findings. The variety of the medical diagnoses in our sample limits the degree to which we can apply our findings to any particular diagnostic group. To address some of these limitations, future studies might include neuropsychological assessments on consecutive admissions to inpatient rehabilitation, focused on more specific medical diagnoses. Other suggestions for future study include comparing neuropsychology raw score models with *z*-score models that adjust for more than age (e.g., education, gender, and race), examining the role of cognition in predicting other outcome variables (e.g., discharge disposition, bowel and bladder continence, and length of rehabilitation stay), and examining other possible predictors of functional ability (e.g., age, presence of depression, and type of cognitive deficit). Lastly, although the model with raw RBANS scores explained almost 30% of the variance in FIM Motor Gain, the majority of the variance remains unexplained.

## 5. Conclusions

This study furthers our understanding of the relationship between cognition and functional ability for patients undergoing inpatient rehabilitation, particularly for those patients who are suspected to have cognitive impairments. Our results provide evidence for the unique contribution of neuropsychological assessment in predicting rehabilitation outcomes. In a regression model, neuropsychological test scores, in combination with the length-of-stay variable, were found to predict improvement in mobility and ADLs for patients undergoing inpatient rehabilitation, in addition to what was predicted by the standard rehabilitation cognitive assessment (Cognitive FIM). Neuropsychology raw scores, unadjusted for age, outperformed age-adjusted scores, providing further evidence of the need to consider absolute cognitive ability when trying to predict performance of real-world skill. If the predictive validity of the models developed in this study is confirmed, this would support the addition of a brief neuropsychological assessment as a source of information about patients' anticipated outcomes at discharge. The neuropsychological findings may also be useful in identifying methods of modifying therapies during the rehabilitation admission in order to maximize rehabilitation improvements for patients with cognitive deficits.

## Figures and Tables

**Table 1:** 

Admitting medical diagnosis	Number of subjects (total *n* = 126)
Stroke (all types)	38
General medical (nonbrain)^1^	23
Brain tumor (all types)	22
Neurological disease (brain)^2^	20
Orthopedic^3^	14
Hydrocephalus (all types)	9

^1^General medical diagnoses included cardiac disease, debility, and spinal disorders. ^2^Neurological diseases included multiple sclerosis, Parkinson's disease, and encephalopathy. ^3^Orthopedic diagnoses included hip fracture, amputations, and multiple-bone fractures.

**Table 2:** Patients referred for neuropsychological assessment, *n* = 126.

RBANS subtest score	Mean *z*-score (percentile)
Figure Copy	−2.32 (1%ile)
Line Orientation	−2.00 (2%ile)
Semantic Fluency	−1.75 (4%ile)
Digit Span	−0.58 (28%ile)
Coding	−3.37 (<1%ile)
List Recall	−1.50 (7%ile)
List Recognition	−2.20 (1%ile)
Story Recall	−1.89 (3%ile)
Figure Recall	−1.60 (5%ile)

**Table 3:** Cognitive test scores predict FIM Motor Gain after contribution of FIM Cognition score.

Model	*F* value	Pr > *F*	*R*-square	AIC
Raw score (FIM Cog, List Recog, Fig Recall, length-of-stay)	11.42	0.0001	0.297	505
*Z*-score (FIM Cog, List Recog, length-of-stay)	6.71	0.0017	0.132	579

List Recog = RBANS List Recognition; length-of-stay = number of rehabilitation days; AIC = Akaike Information Criterion; lower number indicates better model fit; FIM Cog = FIM Admission Cognition; Fig Recall = RBANS Figure Recall.
